# Saikosaponin A attenuates osteoclastogenesis and bone loss by inducing ferroptosis

**DOI:** 10.3389/fmolb.2024.1390257

**Published:** 2024-07-24

**Authors:** Tian-Qi Li, Yan Liu, Chong Feng, Jin Bai, Zi-Rou Wang, Xiang-Yu Zhang, Xin-Xing Wang

**Affiliations:** ^1^ Tianjin Institute of Environmental and Operational Medicine, Tianjin, China; ^2^ School and Hospital of Stomatology, Tianjin Medical University, Tianjin, China

**Keywords:** osteoclasts, lipid peroxidation, ferroptosis, bone diseases, osteolysis, reactive oxygen species

## Abstract

To alleviate bone loss, most current drugs target osteoclasts. Saikosaponin A (Ssa), a triterpene saponin derived from *Bupleurum falcatum* (also known as Radix bupleuri), has immunoregulatory, neuromodulatory, antiviral, anticancer, anti-convulsant, anti-inflammatory, and anti-proliferative effects. Recently, modulation of bone homeostasis was shown to involve ferroptosis. Herein, we aimed to determine Ssa’s inhibitory effects on osteoclastogenesis and differentiation, whether ferroptosis is involved, and the underlying mechanisms. Tartrate‐resistant acid phosphatase (TRAP) staining, F‐actin staining, and pit formation assays were conducted to confirm Ssa-mediated inhibition of RANKL-induced osteoclastogenesis *in vitro*. Ssa could promote osteoclast ferroptosis and increase mitochondrial damage by promoting lipid peroxidation, as measured by iron quantification, FerroOrange staining, Dichloro-dihydro-fluorescein diacetate, MitoSOX, malondialdehyde, glutathione, and boron-dipyrromethene 581/591 C11 assays. Pathway analysis showed that Ssa can promote osteoclasts ferroptosis by inhibiting the Nrf2/SCL7A11/GPX4 axis. Notably, we found that the ferroptosis inhibitor ferrostatin-1 and the Nrf2 activator tert-Butylhydroquinone reversed the inhibitory effects of Ssa on RANKL-induced osteoclastogenesis. *In vivo*, micro-computed tomography, hematoxylin and eosin staining, TRAP staining, enzyme-linked immunosorbent assays, and immunofluorescence confirmed that in rats with periodontitis induced by lipopolysaccharide, treatment with Ssa reduced alveolar bone resorption dose-dependently. The results suggested Ssa as a promising drug to treat osteolytic diseases.

## Graphical Abstract

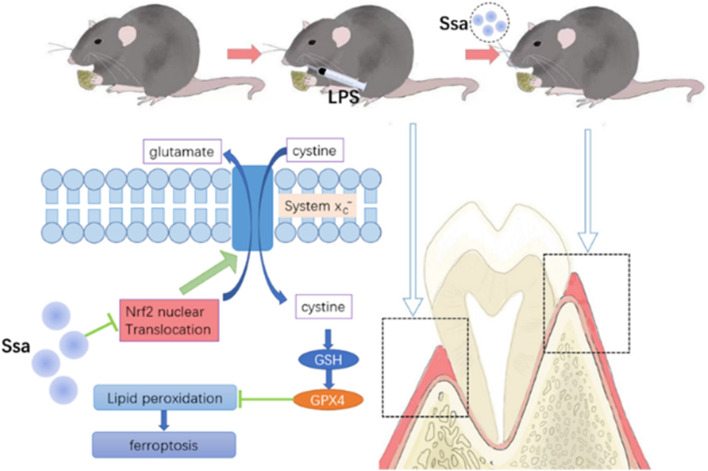

## 1 Introduction

Simultaneous bone resorption and formation are involved in the dynamic process of bone metabolism (Rachner et al., 2011). Consequently, disequilibrium between osteoblasts and osteoclasts can adversely affect bone integrity and its normal function, leading to various pathological lytic bone disorders, including periodontitis, rheumatoid arthritis (RA) and osteoporosis (Pihlstrom et al., 2005; Takayanagi, 2007; Zaidi, 2007). A common feature of these diseases is that osteoclasts are overactivated and bone resorption is enhanced (Boyle et al., 2003). Therefore, it is important to inhibit osteoclast formation during the treatment of lytic bone disorders.

Saikosaponin A (Ssa), a triterpene saponin derived from Bupleurum falcatum, has immunoregulatory, neuromodulatory, antiviral, anticancer, anti-convulsant, anti-inflammatory, and anti-proliferative effects (Ashour and Wink, 2011; Du et al., 2018; Kim, 2018). *In vitro*, Ssa inhibits receptor activator of nuclear factor kappa B ligand (RANKL)-mediated osteoclastogenesis by suppressing the activation of nuclear factor kappa B (NF-κB) and mitogen activated protein kinase (MAPK) (Zhou et al., 2015). However, Ssa’s regulatory mechanisms in osteoclast differentiation and disease are unknown.

Iron-regulated cell death is known as ferroptosis. Ferroptosis, in contrast to other pathways of programmed cell death (e.g., necroptosis and apoptosis), is mainly the result of the intracellular production and degradation of lipid-reactive oxygen species becoming imbalanced under conditions of reduced cellular oxygen resistance and the accumulation of lipid-reactive oxygen species (Hirschhorn and Stockwell, 2019; Li et al., 2021a). Ferroptosis dysregulation is linked to pathological processes, including cancer (Gao et al., 2022), inflammation-related diseases (Stockwell, 2022), and neurodegenerative diseases (Alborzinia et al., 2022). Chang et al. proposed that joint symptoms in patients with arthritis might be relived using ferroptosis inducers (Chang et al., 2022). Wang et al. found that RA pathogenesis and ferroptosis share common characteristics, which suggested that RA might be treated using ferroptosis modulators (Zhao et al., 2022). Qiao et al. demonstrated that lipopolysaccharide (LPS) stimulation of human gingival fibroblasts inflammation involves ferroptosis (Qiao et al., 2022).

Herein, we aimed to determine Ssa’s effects *in vitro* and in an LPS-mediated bone resorption model and investigated the associated molecular mechanisms.

## 2 Materials and methods

### 2.1 Materials and reagents

Amizona Scientific LLC (Birmingham, AL, United States) provided recombinant murine RANKL and macrophage colony stimulating factor (M-CSF), fetal bovine serum (FBS), and minimal essential medium α (α-MEM). Ssa was purchased from MedChemExpress (Shanghai, China). The Tartrate-resistant acid phosphatase (TRAP) Staining kit was obtained from Sigma-Aldrich (St. Louis, MO, United States). The Cell Counting Kit-8 (CCK-8), Lipid Peroxidation Probe -BDP 581/591 C11, Iron Assay Kit-Colorimetric kit, and FerroOrange were obtained from Dojindo Molecular Technologies (Kumamoto, Japan). Thermo Fisher Scientific (Waltham, MA, United States) provided the MitoSOX Red mitochondrial superoxide indicator. Primary antibodies against nuclear factor erythroid 2-related factor 2 (Nrf2), solute carrier family seven member 11 (SCL7a11), cathepsin K and glutathione peroxidase 4 (GPX4) were purchased from HUABIO (Hangzhou, China). Antibodies against Fos proto-oncogene, AP-1 transcription factor subunit (c-Fos), TNF receptor associated factor 6 (TRAF6), and β-actin were obtained from Abcam (Cambridge, United Kingdom. Bioworld (Nanjing, China) provided the goat-anti-rabbit and secondary antibodies. The enzyme-linked immunosorbent assay (ELISA) kits for osteoprotegerin (OPG), ACP5, and the alkaline phosphatase (ALP) biochemical kits were obtained from Nanjing Jiancheng (Nanjing, China). Beyotime (Shanghai, China) provided the remaining reagents and kits.

### 2.2 Cell culture and differentiation of osteoclasts

C57BL/6 mice (six to eight weeks old) were sacrificed, and their long bones were removed, to isolate bone marrow-derived macrophages (BMMs). Soft tissues were removed from mice’s femurs and tibias. After flushing the bone marrow cavities of the long bones using α-MEM containing 10% FBS, we isolated bone marrow suspensions. The next day, cells were separated from the suspension and then the erythrocytes were lysed. The nonadherent cells (BMMs) were cultured in α-MEM for subsequent use. M CSF(40 ng/mL) was used in all experiments to maintain BMMs survival. To stimulate osteoclast differentiation, 50 ng/mL of RANKL was administered. Next, 4% formalin was used to fix the cells for 30 min, followed by TRAP staining. We identified osteoclasts as TRAP positive cells with > three nuclei.

### 2.3 Assay for cell viability

According to the manufacturer’s instructions, cell viability was measured using a CCK-8 kit. 5 × $10^{4}$ cells were inoculated into a 96-well plates and grown for 24 h. Subsequently, various doses of Ssa (3.125, 6.25, 12.5, and 25 µM) were used to treat the cells for 96 h, with or without RANKL. To analyze the cause of cell death, BMMs were administered with 10 μM of ZVAD-FMK, 10 μM of necrostatin-1, and 2 μM of Ferrostatin-1 (Fer-1), for 96 h, with or without Ssa (6.25 μM). CCK-8 (10 μL) was added and incubated for 1.5 h at 37°C. A microplate reader was then used to measure the absorbance at 450 nm.

### 2.4 Formation of the F-actin ring

BMMs were grown in medium comprising 40 ng/mL of M-CSF, 50 ng/mL of RANKL, and different amounts of Ssa. Four days later, mature osteoclasts were identified, followed by fixation for 15 min. The cells were incubated with Actin-Tracker Red Rhodamine to visualize the osteoclast cytoskeletons and 4, 6-diamidino-2-phenylindole (DAPI) was used to identify nuclei. A fluorescence microscope was then used to image the F-actin rings.

### 2.5 Resorption pit assay

BMMs were seeded on bone slices and stimulated with M-CSF and RANKL for 10 days, with or without Ssa (6.25 and 12.5 μM). A scanning electron microscope (ZEISS GeminiSEM 300, Oberkochen, Germany) was employed to view the effect the BMMs on the bovine bone slices.

### 2.6 Iron quantification and ferroOrange staining

Based on the instructions provided by the manufacturer, an iron assay kit was used to determine the intracellular iron concentration. The FerroOrange fluorescent probe enables live-cell fluorescent imaging of intracellular Fe2+. The cells were added with medium without FBS, but with 1 μmol/L of the FerroOrange reagent. Following incubation for 30 min, a fluorescence microscope was used to visualize the intracellular iron ions.

### 2.7 Mitochondrial and intracellular and reactive oxygen species (ROS) determination

Dichloro-dihydro-fluorescein diacetate (DCFH-DA) and MitoSOX™ Red were used to detect the intracellular and mitochondrial ROS, respectively. Cells were incubated with DCFH‐DA in a 37°C incubator for 30 min or with MitoSOX™ at 37°C for 10 min. Following three washes with 1 × phosphate-buffered saline (PBS), a microscope was used to view the cells.

### 2.8 Malondialdehyde (MDA) and glutathione (GSH) contents

A Lipid Peroxidation MDA assay kit was used to determine the MDA content in cell lysates, and the absorbance at 532 nm was measured using a microplate reader. BMM GSH contents were assayed using a GSH and GSSG Assay Kit following the supplier’s protocol. The total protein level, as assessed using a BCA Protein Assay Kit, was used to normalize the MDA and GSH assay results.

### 2.9 Lipid peroxidation assay

Lipid peroxidation was detected using boron-dipyrromethene (BODIPY™) 581/591 C11 (2 μmol/L), which was incubated directly with cells for 30 min at 37°C. Fluorescence was detected using a confocal microscope.

### 2.10 JC-1 fluorescence assay

JC-1 fluorescence mitochondrial imaging was used to determine the mitochondrial membrane potential. JC-1 was incubated with the cells 20 min at 37°C. The cells were then washed twice using JC-1 buffer. The fluorescent images were captured using a laser scanning confocal microscope. The mitochondrial membrane potential was represented by the ratio of red to green fluorescence.

### 2.11 Quantitative real-time reverse transcription PCR (qRT-PCR)

The TRIzol reagent was used to isolate total RNA according to the supplier’s guidelines and reverse transcribed to cDNA employing a Prime Script RT-PCR kit (TAKARA Korea, Seoul, Korea). The cDNA was used as the template for a quantitative real-time PCR reaction carried out using SYBR mix in the CFX384 real-time system (BioRad, Hercules, CA, United States). The primers utilized were synthesized by Sangon Biotech (Shanghai, China) and are shown in Table 1.

**TABLE 1:** Primer sequences for real-time PCR.

Gene	Forward primer, 5′ to 3′	Reverse primer, 5′ to 3′
Ctsk	GAA​GAA​GAC​TCA​CCA​GAA​GCA​G	TCC​AGG​TTA​TGG​GCA​GAG​ATT
*Acp5*	CAC​TCC​CAC​CCT​GAG​ATT​TGT	CAT​CGT​CTG​CAC​GGT​TCT​G
Nfatc1	GAC​CCG​GAG​TTC​GAC​TTC​G	TGA​CAC​TAG​GGG​ACA​CAT​AAC​TG
*Fos*	TTG​GCA​CTA​GAG​ACG​GAC​AGA	CGG​GTT​TCA​ACG​CCG​ACT​A
Actb	GGC​TGT​ATT​CCC​CTC​CAT​CG	CCA​GTT​GGT​AAC​AAT​GCC​ATG​T

### 2.12 Western blotting

Cell lysis buffer was used to lyse the cells, whose total protein concentration was quantified using the BCA protein assay kit. 4%–12% SDS-PAGE was used to separate the proteins in the cell lysate, which were eletrotransferred onto polyvinylidene fluoride membranes. 5% non-fat milk was used to block the membranes for 2 h, followed by incubation with the primary antibodies overnight at 4°C. Next day, the membranes were reacted with anti-rabbit secondary antibody conjugated with horseradish peroxidase (HRP) (1:10,000) for 1 h at room temperature. Finally, an Amersham Imager 680 image system (Amersham United Kingdom) was used to capture the immunoreactive protein signals.

### 2.13 Immunofluorescence staining

Cells were washed three time using PBS, fixed using 4% paraformaldehyde at room temperature for 30 min, blocked using 5% (w/v) BSA in PBS-Tween20 (PBST), and then immunoassayed using anti-cathepsin K (CTSK), anti-Nrf2, and anti-c-FOS antibodies at 4°C overnight. Next day, the cells were reacted with goat anti-rabbit Alexa Fluor-488-conjugated secondary antibody, washed three times with PBS, DAPI-stained, and observed under a laser scanning confocal microscope.

### 2.14 LPS-induced bone resorption model

The Institutional Animal Ethics Committee supervised all the processes and approved the animal experiments. Twenty 10-week-old Wistar rats were divided randomly into four groups of 5: Sham group, LPS group, and LPS + Ssa at low concentration (50 mg/kg body weight) and high concentration (100 mg/kg body weight). Every other day, we injected normal saline into the Sham group, and injected LPS into the other groups, with or without Ssa administration by gavage. After 4 weeks of treatment, all rats were euthanized. Their alveolar bones were fixed for micro-computed tomography (µCT) and histological analysis, and their collected serum was used for biochemical assays and ELISA.

Three-dimensional images of the calvaria were scanned using a high resolution µCT scanner following the supplier’s guidelines.

### 2.15 Histological analysis

The alveolar bones of the rats in each group were decalcified, paraffin embedded, sectioned using a microtome at 4 μm thick, and stained using hematoxylin and eosin (H&E) and TRAP for osteoclasts. Immunofluorescence was used to show protein expression in the cells.

### 2.16 Statistical considerations

Data from the experiments are displayed as the mean ± SD (*n* ≥ 3). Student’s *t*-test between the treatment and control groups or analysis of variance (ANOVA) for multiple groups were used to determine the statistical significance. A *p*-value less than 0.5 was considered to indicate statistical significance.

## 3 Results

### 3.1 Ssa inhibits the osteoclastogenesis induced by RANKL *in vitro*

**FIGURE 1:**
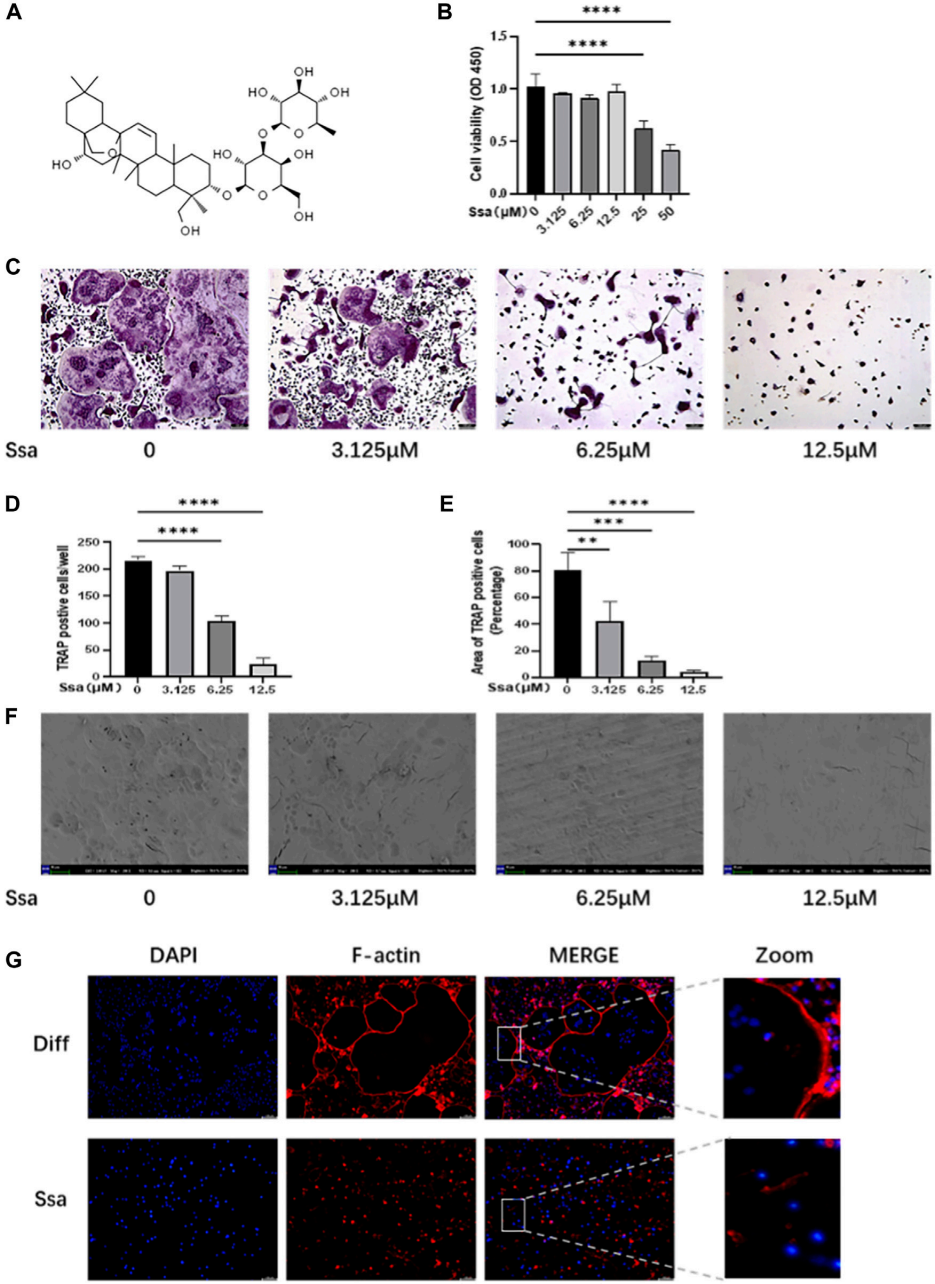
Ssa inhibits RANKL-induced osteoclastogenesis *in vitro.* **(A)** The chemical structural formula of Ssa. **(B)** BMMs were treated with different concentrations of Ssa for 96 h and a CCK8 assay was used to test the cell Viability. **(C)** Cultures of RANKL and M-CSF-incubated BMMs and treated using various concentrations of Ssa for 4 days, followed by TRAP staining. Scale bars = 100 μm. **(D)** Osteoclast were enumerated under a microscope. **(E)** ImageJ software was used to determine the osteoclast areas. **(F)** Assay for osteolytic function involving inoculating BMMs on finished cow bone fragments followed by scanning electron microscopy imaging. Scale bars = 500 μm. **(G)** To assess the impact of Ssa on mature osteoclast generation, we carried out F-Actin ring formation assays. Scale bars = 100 μm. In the graphs, data are displayed as the mean ± SD (*n* = 3). **p < 0.01; ***p < 0.001, and ****p < 0.0001. Ssa, Saikosaponin A; BMMs, bone marrow macrophages; CCK8, Cell Cycle Kit-8; RANKL, receptor activator of nuclear factor kappa B ligand; M-CSF, macrophage colony stimulating factor; TRAP, tartrate-resistant acid phosphatase; DAPI, 4′,6-diamidino-2-phenylindole; CTRL, control.

The toxicity of Ssa toward primary BMMs was assessed. CCK-8 assays suggested that there was no detectable toxicity of Ssa toward BMMs until the concentration reached 12.5 μM (Figures 1A,B). To determine the effect of Ssa on the differentiation of RANKL-induced osteoclasts, BMMs incubated with RANKL and M-CSF were treated with different Ssa concentrations (0, 3.125, 6.25, and 12.5 μM). TRAP staining showed that Ssa markedly inhibited multinucleated osteoclast differentiation in a dose-dependent manner (Figure 1C). As the concentration of Ssa increased, the density and coverage of multinucleated TRAP-positive osteoclasts gradually decreased (Figures 1D,E). Next, the effect of Ssa on bone-resorbing activity was examined, which showed that that Ssa dose-dependently inhibited bone resorption (Figure 1F). Moreover, since F-actin ring formation is neceSsary for osteoclast function, we tested this process using phalloidin. Immunofluorescent staining demonstrated that Ssa markedly inhibited typical F-actin ring formation relative to that in the control group (Figure 1G). The findings clearly demonstrated that Ssa inhibits osteoclast function and bone-resorbing activity *in vitro*.

### 3.2 Osteoclast-specific gene expression is inhibited by Ssa

**FIGURE 2:**
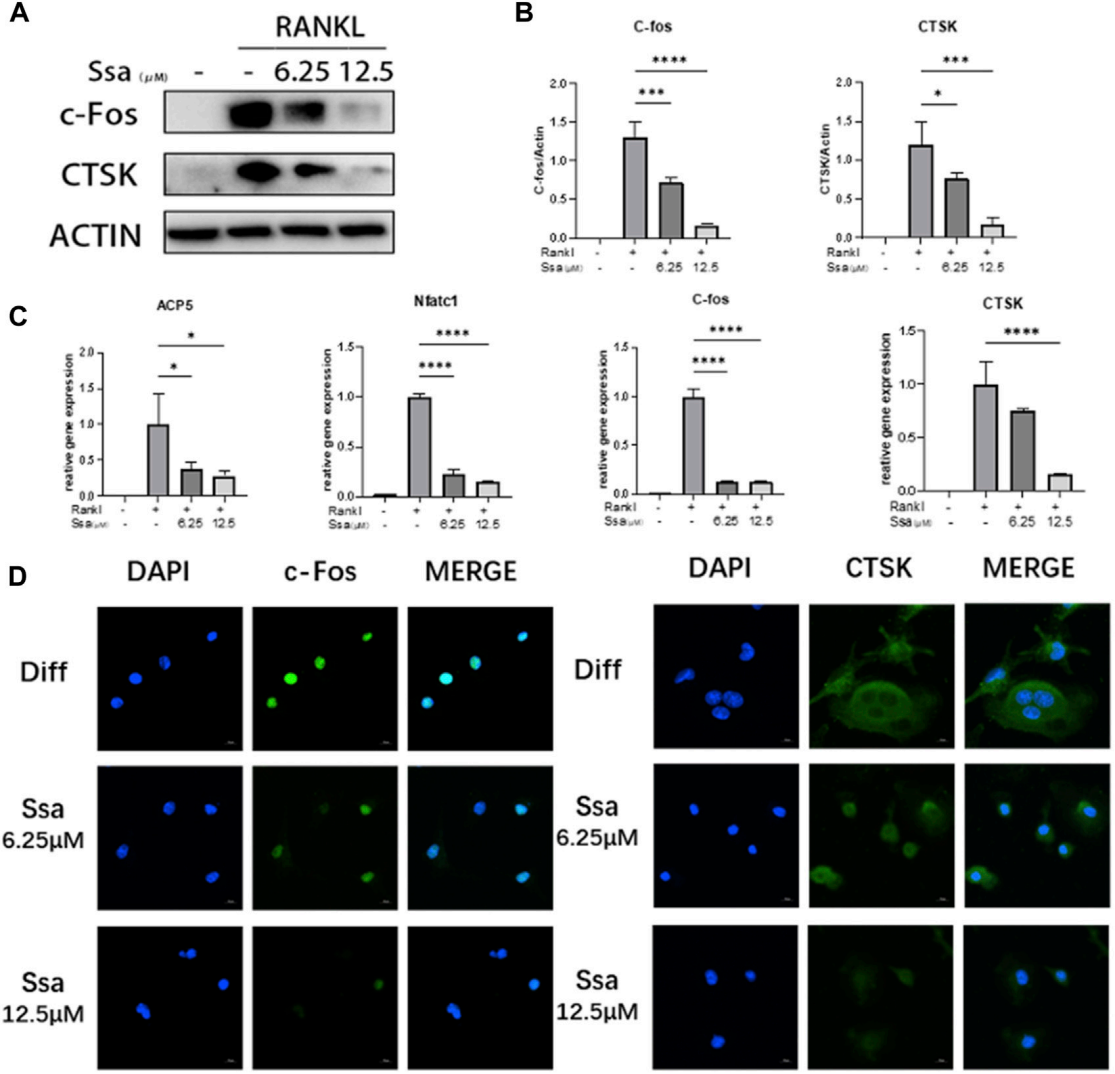
Osteoclast-specific gene expression was inhibited by Ssa. **(A)** BMMs were cultured with different doses of Ssa (0, 6.25, and 12.5 μM) for 4 days. The protein levels of c-Fos and CTSK were analyzed using Western blotting. **(B)** Quantitative analysis of the concentration dependence of osteoclast associated proteins. **(C)** qRT-PCR determination of *Acp5*, *Nfatc1*, *Ctsk*, and *Fos* mRNA expression. **(D)** Immunofluorescence detection of CTSK and c-Fos in BMMs after 4 days of RANKL stimulation. The concentration of Ssa was 6.25 μM. Scale bars = 10 μm. In the graphs, data are displayed as the mean ± SD (*n* = 3). *p < 0.05; ***p < 0.001, and ****p < 0.0001. c-Fos, Fos proto-oncogene, AP-1 transcription factor subunit; CTSK, cathepsin K; ACP5, acid phosphatase 5, tartrate resistant; NFATC1, nuclear factor of activated T cells 1.

We further assessed whether Ssa inhibited the expression of osteoclast‐related genes and proteins. Western blotting demonstrated that the levels of proteins associated with osteoclast differentiation (cathepsin K (CTSK) and c-Fos) were in a dose-dependent manner after Ssa intervention (Figures 2A,B). qRT‐PCR analysis displayed that the mRNA expression levels of genes related to the functions of mature osteoclast, including *Acp5* (encoding acid phosphatase 5, tartrate resistant), *Nfatc1* (encoding nuclear factor of activated T cells 1), *Ctsk* and *Fos* increased markedly at 4 days after RANKL intervention relative to those in the undifferentiated cells, which were decreased in the Ssa-treated groups, especially when the dose is higher (Figure 2C). Immunofluorescence verified that Ssa markedly decreased the expression of c-Fos and CTSK (Figure 2D).

### 3.3 Ssa promotes osteoclasts ferroptosis by inhibiting the Nrf2/SCL7A11/GPX4 signaling pathway

**FIGURE 3:**
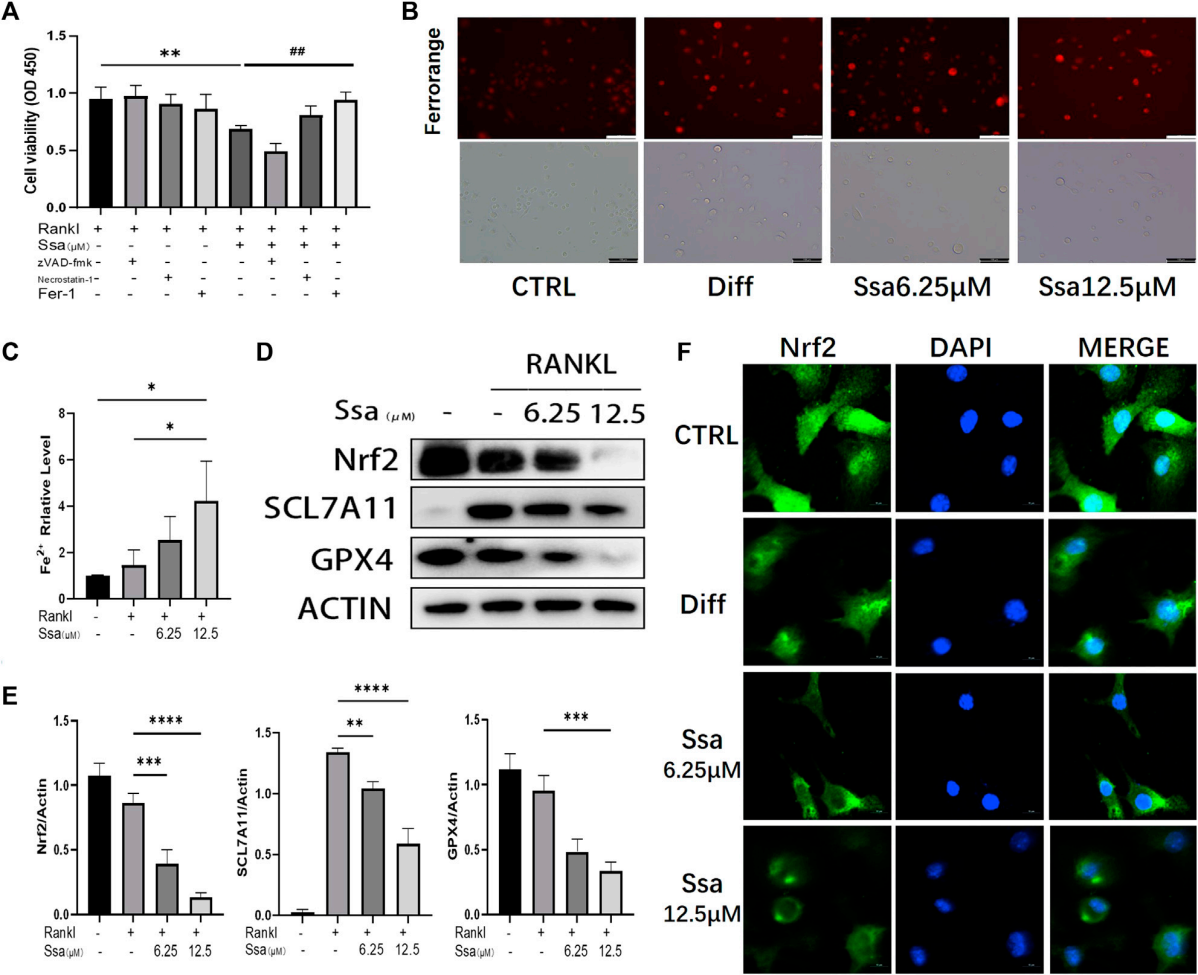
Ssa promotes osteoclasts ferroptosis by inhibiting the Nrf2/SCL7A11/GPX‐4 pathway. **(A)** Effect of different inhibitors in the presence of RANKL on cell death of BMMs induced by Ssa. The following inhibitors were used: 10 μM zVAD-fmk, 10 μM Necrostatin-1s, and 1 μM Ferrostain-1. **(B)** Intracellular Fe^2+^ detected by FerroOrange. Scale bars = 100 μm. **(C)** Intracellular iron content was tested using ELISA. **(D)** Quantitative Western blotting analysis of SLC7A11, GPX4, and Nrf2 protein levels **(E)**. **(F)** Nrf2 nuclear translocation detected using immunofluorescence staining. Scale bars = 10 μm. In the graphs, data are displayed as the mean ± SD (*n* = 3). *p < 0.05; **p < 0.01; ##*p* < 0.01; ***p < 0.001 and ****p < 0.0001. Nrf2, nuclear factor erythroid 2-related factor 2; SCL7A11, solute carrier family seven member 11; GPX4, glutathione peroxidase 4; ELISA, enzyme-linked immunosorbent assay; Fer-1, Ferrostatin-1.

To investigate the impact of Ssa on osteoclast differentiation, BMMs were induced using RANKL and treated with or without Ssa (6.25 μM) for 4 days. Ssa treatment suppressed the viability of RANKL-treated cells. Ferrostatin-1 (a selective ferroptosis inhibitor), Necrotatin-1 (a specific necroptosis inhibitor), and zVAD-fmk (a specific apoptosis inhibitor) were employed to study the mechanism by which Ssa inhibits osteoclast differentiation. Impressively, the CCK-8 assay results showed that ferrostatin-1, but not Necrostatin-1 or zVAD-fmk, markedly inhibited osteoclast death induced by Ssa (Figure 3A). The FerroOrange fluorescent probe was employed to detect the level of ferroptosis. Ssa markedly increased the red fluorescence density (representing the Fe2+ content) compared with that in Control group (Figure 3B). The levels of Fe2+ in cells were also assessed using iron assay kits, which confirmed the results above (Figure 3C). GPX4 is an important ferroptosis regulator, the upstream mediator of which is SLC7A11 (Zhang et al., 2021). Moreover, Nrf2 regulates SLC7A11 to inhibit ferroptosis (Xie et al., 2020; Yuan et al., 2021). Therefore, to determine whether ferroptosis is associated with Ssa-mediated inhibition of RANKL-induced osteoclastogenesis in BMMs, the protein levels of SLC7A11, GPX4, and Nrf2 were determined. Ssa (6.25 μM) markedly reduced Nrf2, SLC7A11, and GPX4 levels in BMMs (Figures 3D,E). Simultaneously, Ssa treatment strikingly reduced Nrf2 nuclear translocation (Figure 3F). Therefore, we concluded that Ssa regulates the Nrf2/SLC7A11/GPX4 axis to promote the ferroptosis of osteoclasts.

### 3.4 Ssa increases mitochondrial damage by promoting lipid peroxidation

**FIGURE 4:**
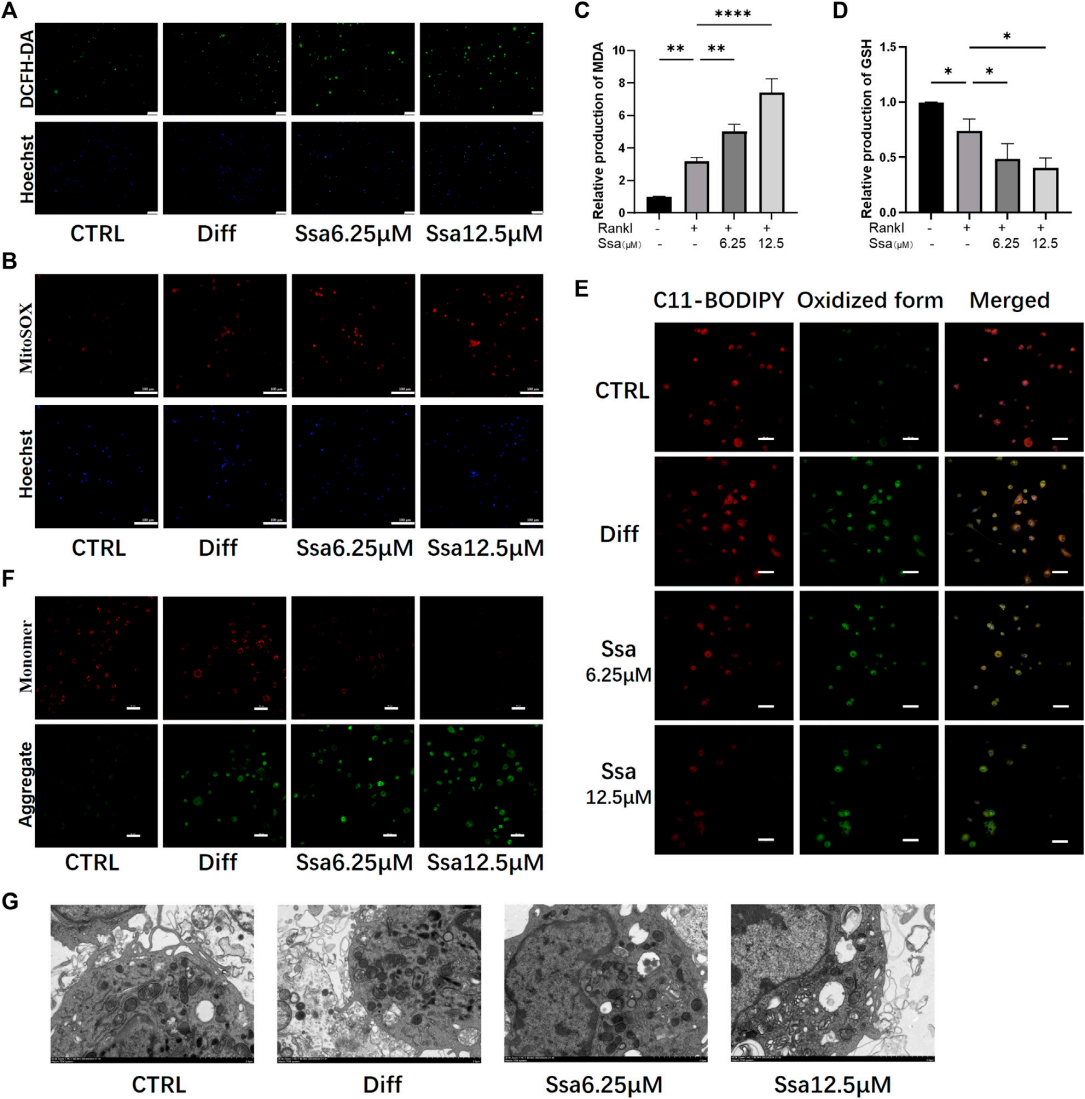
Ssa increases mitochondrial damage by promoting lipid peroxidation. **(A)** DCFH-DA. Original scale bars: 100 μm. **(B)** MitoSOX. Original scale bars: 100 μm. **(C)** MDA. **(D)** GSH. **(E)** BODIPY 581/591 C11. Original scale bars: 20 μm. **(F)** JC-1. Scale bars = 50 μm.**(G)** Under the transmission electron microscope, Ssa treatment caused mitochondrial shrinkage, enhanced density of the mitochondrial membrane, density, and mitochondrial cristae disappearance. In the graphs, data are displayed as the mean ± SD (*n* = 3). *p < 0.05; **p < 0.01, and ****p < 0.0001. DCFH-DA, Dichloro-dihydro-fluorescein diacetate; MDA, malondialdehyde; GSH, glutathione; BODIPY, boron-dipyrromethene.

Ferroptosis and ROS accumulation are closely related (Lin and Beal, 2006; Dixon et al., 2012); therefore, we assessed total ROS production using DCFH‐DA. RANKL significantly increased ROS levels in BMMs, and further Ssa treatment increased the ROS levels to a greater extent than RANKL treatment alone (Figure 4A). MitoSox‐Red staining showed that the mitochondrial ROS levels were consistent with the changes in intracellular ROS abundance. Collectively, these findings indicated that ROS are crucial for Ssa‐induced ferroptosis (Figure 4B). Next, the levels of oxidative stress-related factors MDA and GSH were detected. Ssa intervention could increase MDA levels (Figure 4C), and decrease GSH levels (Figure 4D). Next, we used BODIPY 581/591 C11 (Figure 4E) and the JC‐1 probe (Figure 4F) to detect the levels of lipid peroxidation and mitochondrial membrane potential (MMP), respectively. The analysis indicated that lipid peroxides accumulated and the MMP increased after the addition of RANKL. The addition of Ssa caused a further increase. Ssa-treated cells exhibited the characteristic morphological features of ferroptosis, including a reduction in mitochondrial membrane density and corresponding volume, diminished or vanished mitochondrial cristae, and a rupturing of the outer membrane (Figure 4G).

### 3.5 Ferroptosis is involved in Ssa inhibition of osteoclast differentiation

**FIGURE 5:**
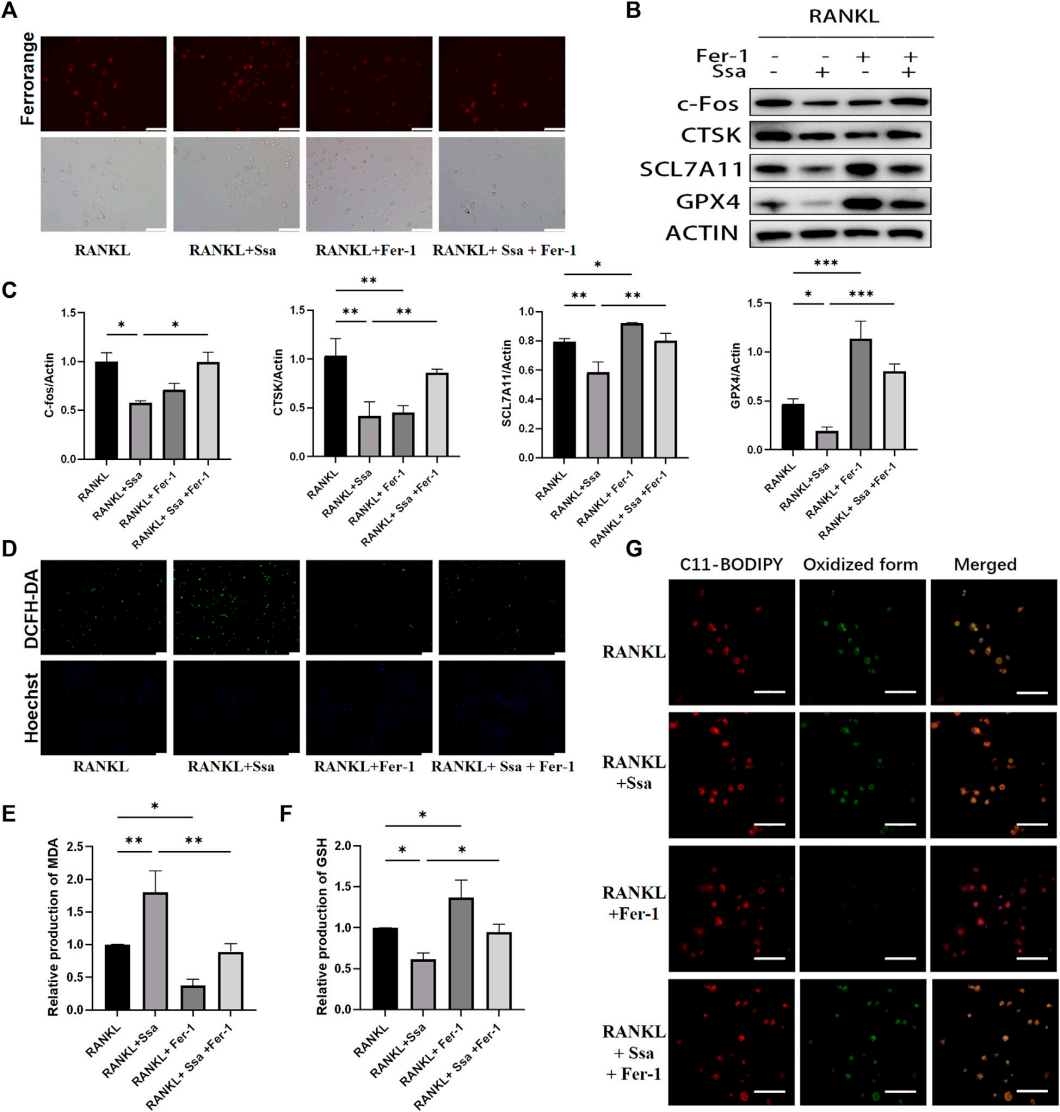
Ferroptosis is involved in Ssa inhibition of osteoclast differentiation. **(A)** FerroOrange staining. Scale bars = 100 μm. **(B–C)** Western blotting assay of CTSK, c- Fos, Nrf2, SLC7A11, and GPX4 levels and their quantification. **(D)** DCFH-DA. Scale bars = 100 μm. **(E)** MDA. **(F)** GSH. **(G)** BODIPY 581/591 C11. Scale bars = 20 μm. In the graphs, data are displayed as the mean ± SD (*n* = 3). *p < 0.05; **p < 0.01 and ***p < 0.001.

The ferroptosis inhibitor ferrostatin-1 was administered to further verify above conclusion. BMMs were classified into four groups: The RANKL group, the RANKL + Ssa group, the RANK + Fer-1 group, and the RANKL + Ssa + Fer-1 group. The RANKL + Ssa + Fer-1 group showed markedly increased Fe2 + levels compared with those in the RANK + Ssa group (Figure 5A). The levels of ferroptosis-associated proteins GPX4, SLC7A11, and Nrf2 increased markedly and the levels of osteoclast differentiation proteins CTSK and c-Fos decreased significantly (Figures 5B,C). ROS levels in the RANKL + Ssa + Fer-1 group increased markedly relative to those in the RANKL + Ssa group (Figure 5D). The MDA level increased and the GSH level decreased in the RANK + Ssa + Fer-1 group compared with those in the RANKL + Ssa group (Figures 5E,F). Subsequently, the amount of lipid peroxides in the RANKL + Ssa + Fer-1 group was increased markedly significantly increased relative to that in the RANKL + Ssa group (Figure 5G). These results showed that ferrostatin-1 reversed the inhibitory effect of Ssa on RANKL-induced osteoclastogenesis, suggesting that Ssa inhibits RANKL-induced osteoclastogenesis by promoting ferroptosis.

### 3.6 Activating Nrf2 attenuates Ssa-induced osteoclasts ferroptosis

**FIGURE 6:**
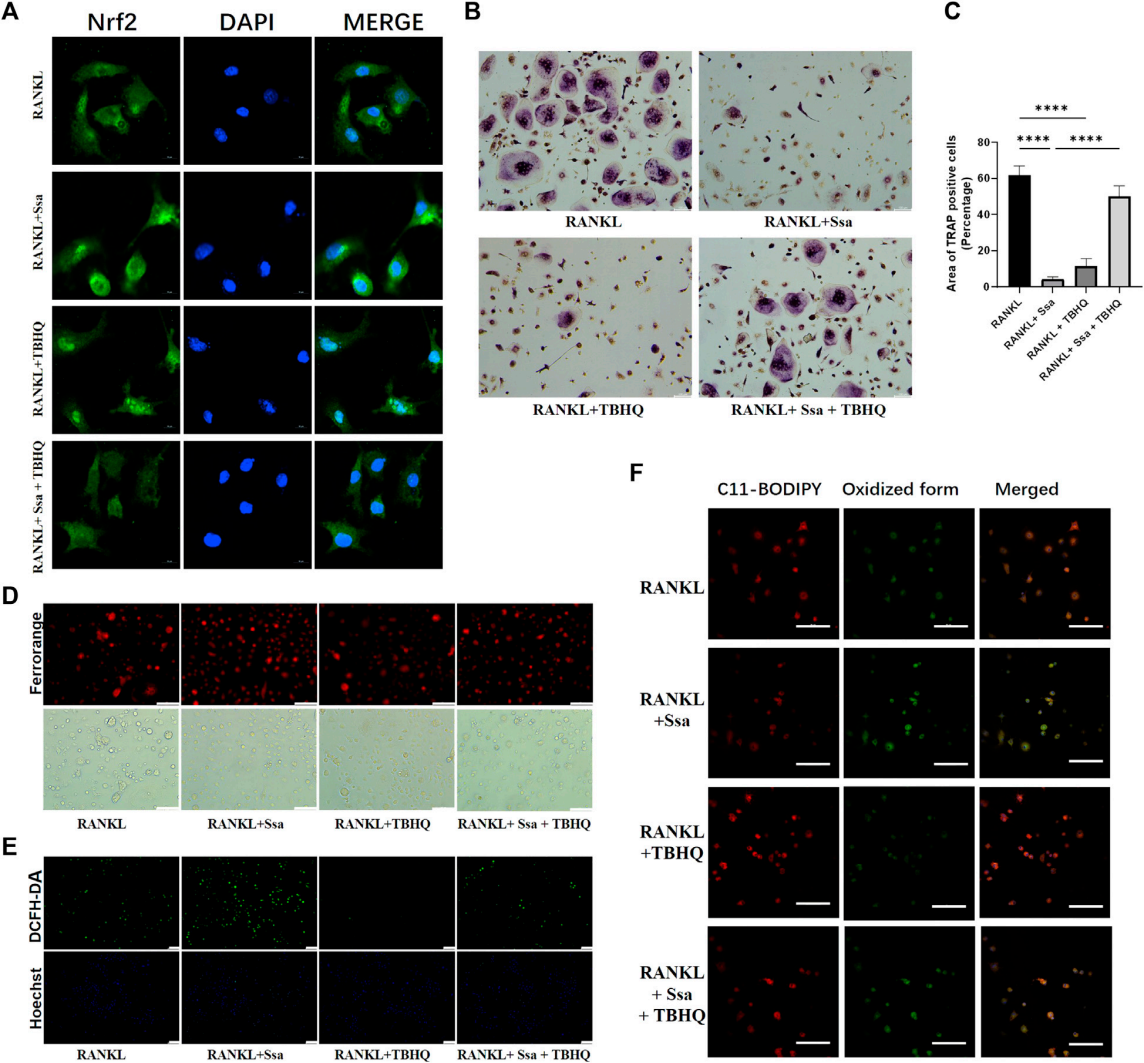
Activating Nrf2 attenuates Ssa-induced osteoclast ferroptosis. **(A)** Nrf2 nuclear translocation detected using immunofluorescence staining. Scale bars = 10 μm. **(B)** TRAP staining. Scale bars = 100 μm. **(C)** Enumeration of osteoclast under a microscope. **(D)** FerroOrange. Scale bars = 100 μm. **(E)** DCFH-DA. Scale bars = 100 μm. **(F)** BODIPY 581/591 C11. Scale bars = 50 μm. In the graphs, data are displayed as the mean ± SD (*n* = 3). ****p < 0.0001.

The Nrf2 activator TBHQ was used to confirm the role of the Nrf2 signaling pathway in the ferroptosis induced by Ssa in osteoclasts. Immunofluorescence assay of Nrf2 expression confirmed that TBHQ significantly increased the fluorescence signal of Nrf2 (Figure 6A). TRAP staining demonstrated markedly increased osteoclastogenesis in the RANKL + Ssa + TBHQ relative to that in the RANKL + Ssa group (Figures 6B,C). Compared with that in the RANKL + Ssa group, the iron and ROS contents decreased in the RANKL + Ssa + TBHQ group (Figures 6D,E). Fluorescence detection of the lipid peroxide products showed that the RANKL + Ssa group had lower levels of lipid peroxide products after TBHQ treatment (Figure 6F). Collectively, these results revealed that Ssa could promote osteoclast ferroptosis by inhibiting the Nrf2/SCL7A11/GPX4 signaling pathway, which attenuated alveolar bone resorption in the rat model of periodontitis.

### 3.7 Ssa attenuates bone loss in experimental periodontitis

**FIGURE 7:**
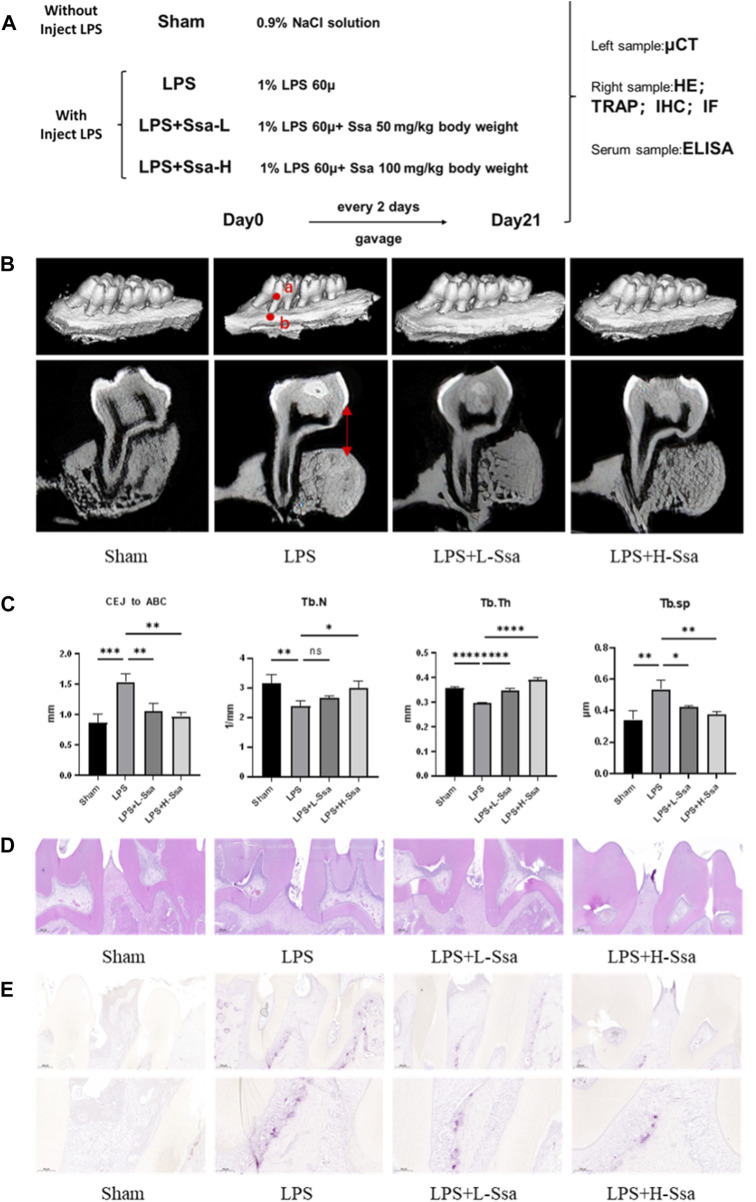
Ssa attenuates bone loss in experimental periodontitis. **(A)** Design of the experiment. **(B)** μCT reconstruction of the alveolar bone. The distance from the CEJ to the ABC is indicated using a red arrow. **(C)** Quantitative analysis of the CEJ-ABC distance, and parameters of the bone trabecula: trabecular number (Tb.N), trabecular thickness (Tb.Th) and trabecular separation (Tb.Sp). **(D)** Histopathological analysis of periodontal lesions. Scale bars = 200 μm. **(E)** TRAP staining results. Scale bars = 200 μm and 100 μm. In the graphs, data are displayed as the mean ± SD (*n* = 3). *p < 0.05; **p < 0.01; ***p < 0.001 and ****p < 0.0001. Ssa, Saikosaponin A; μCT; micro computed tomography; CEJ, cementoenamel junction; ABC, alveolar bone crest (ABC); LPS, lipopolysaccharide; HE, hematoxylin and eosin; IHC, immunohistochemistry; IF, immunofluorescence.

**FIGURE 8:**
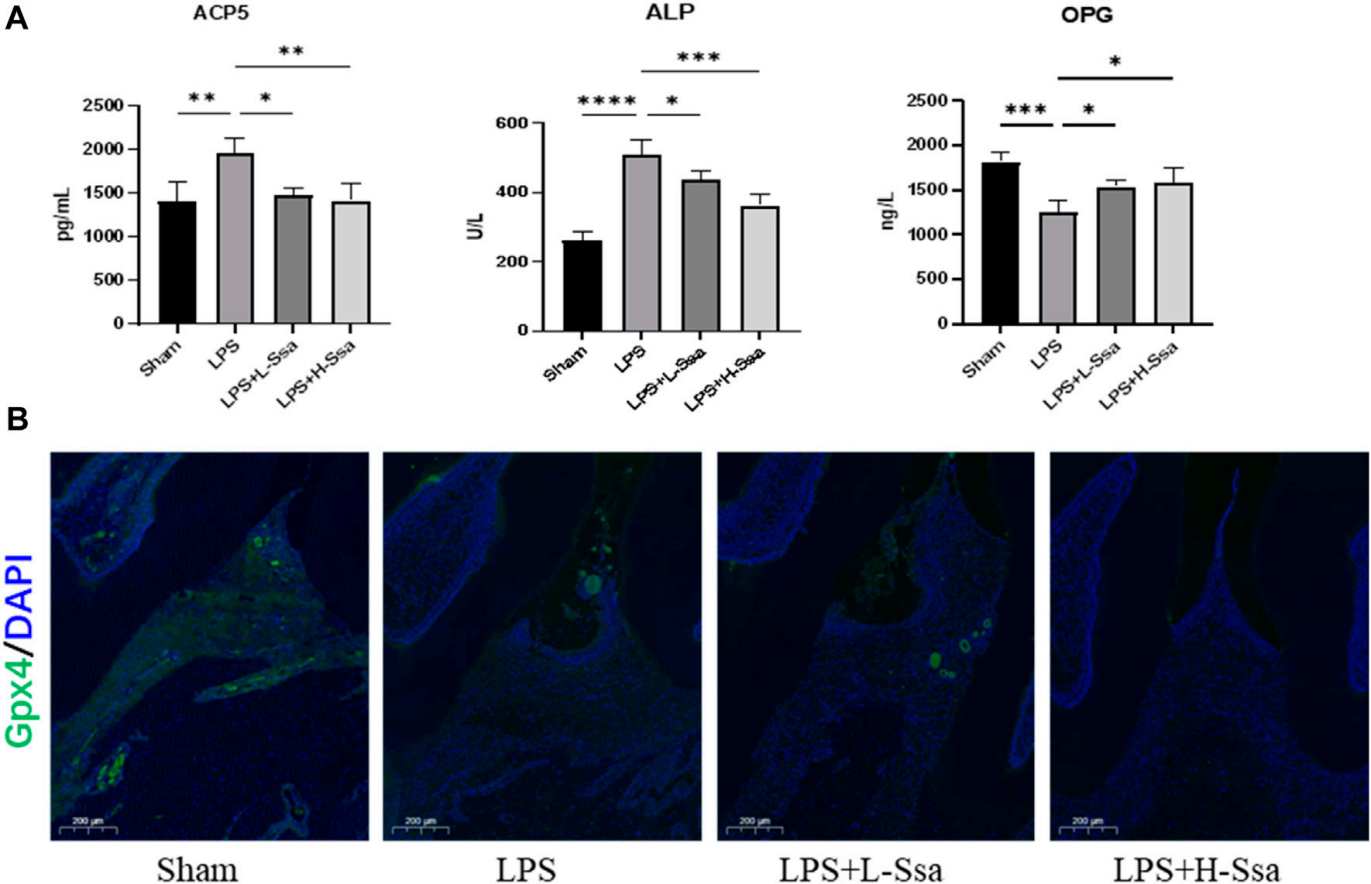
Ssa attenuates bone loss by inhibiting the Nrf2/SCL7A11/GPX4 signaling pathway without osteoblast toxicity. **(A)** ACP5, ALP, and OPG levels in serum. **(B)** Immunofluorescence analysis of GPX4 expression. Scale bars = 200 μm. OPG, osteoprotegerin.

To determine whether Ssa could alleviate LPS-mediated bone resorption, a rat model of periodontitis was induced using LPS (illustrated diagrammatically in Figure 7A). The reconstructed 3D model overall morphology and palatal linear bone loss are shown in Figure 7B. The LPS group had the greatest extent of alveolar bone loss. Compared with that in the Sham group, the distance between the cementoenamel junction (CEJ) and the alveolar bone crest (ABC) for the first molar increased markedly relative to that in the Sham group, thus verifying the successful construction of the experimental model of periodontitis. Ssa decreased the CEJ–ABC distances. CT analysis showed that model group had marked bone loss, accompanied by decreased thickness (Tb.Th) and the trabecular number (Tb.N), together with increased trabecular spaces (Tb.Sp) relative to that in the LPS group. Compared with the model group, Ssa treatment reduced alveolar erosion and reversed the trabecular parameters. The treatment effect in the high-dose Ssa group was better than that of low-dose Ssa group (Figure 7C). H&E staining revealed that compared with the Sham group, the attachment of collagenous fibers was destroyed and the inflammatory cell infiltration into the epithelial layer and connective tissue was significantly reduced in the groups treated with intragastric administration of Ssa (Figure 7D). In addition, TRAP staining revealed an increase in the number of large osteoclasts in the Sham group compared with the reduced osteoclast formation in the LPS + Ssa group (Figure 7E). Serum indicators showed significantly increased ACP5 and ALP levels in the LPS group, suggesting enhanced osteoclastogenesis and repair of osteogenesis, meanwhile the levels of OPG, an osteoclast suppressive cytokine, were decreased. Ssa treatment effectively reversed the levels of these biomarkers compared with those in the LPS group (Figure 8A). Moreover, Ssa caused a decrease in GPX4 on the inflammatory alveolar bone surfaces (Figure 8B). Thus, Ssa could decrease osteoclastogenesis to attenuate alveolar bone resorption in the rat model of periodontitis and attenuated bone loss by inhibiting the Nrf2/SCL7A11/GPX4 signaling pathway without osteoblast toxicity.

## 4 Discussion

The proliferation and activation of osteoclasts is promoted by pathological bone homeostasis, resulting in substantial disruption of bone, forming a common mechanism in a number of osteolytic diseases (Bertuglia et al., 2016; Mukherjee and Chattopadhyay, 2016; Kapasa et al., 2017). It was proposed that these disease could be treated using therapies that inhibit osteolysis (Hadji et al., 2015; Li et al., 2021b). Considering the side effects of several of these treatments (e.g., muscle and joint pain and gastrointestinal symptoms, (Hough et al., 2014; Compston et al., 2019; Sun X. et al., 2020), developing treatments that attenuate osteoclastogenesis but lack side-effects is imperative. Historically, natural products have provided various therapeutic agents and lead drugs (Koehn and Carter, 2005). Recently, researchers have sought small bioactive molecules derived from natural products that can prevent, treat, or both, pathological osteopenic diseases without deleterious side effects.

Ssa shows anti-inflammatory activity (Gao et al., 2017), and reduced inflammation and oxidative stress in human umbilical vein endothelial cells stimulated with LPS (Fu et al., 2015). Furthermore, Ssa could attenuate T cell activation and proliferation via cell cycle arrest and apoptosis induction (Sun et al., 2009). *In vitro*, Ssa could attenuate osteoclastogenesis induced by RANKL by decreasing MAPK and NF-κB activation (Hadji et al., 2015). However, the regulatory mechanisms of Ssa in osteoclast differentiation and osteolytic disease are poorly understood. Herein, our findings showed that Ssa inhibited *in vitro* osteoclastogenesis and reduced *in vivo* bone loss induced by LPS by promoting osteoclast ferroptosis. Moreover, Ssa-promoted ferroptosis was partially induced by Nrf2/SLC7A11/GPX4 signal pathway inactivation. *In vitro* and *in vivo*, Ssa did not affect osteoblast differentiation (Supplementary Figure S1). Thus, the application of Ssa represents an appealing strategy to treat a series of pathological lytic bone disorders.

Ferroptosis is recently discovered type of cell death involving iron accumulation and iron-mediated lipid peroxidation (Tang et al., 2021). The system xc-/GSH/GPX4 axis has a crucial function to prevent ferroptosis induced by lipid peroxidation (Chen et al., 2021). Furthermore, the pathophysiological relevance of ferroptosis, especially as a therapeutic modality in cancer and ischemic organ damage, has been convincingly established (Jiang et al., 2021). Iron metabolism regulation involves several mechanisms, such as endoplasmic reticulum stress, oxidative stress, and ferritin autophagy (Chen et al., 2020; Li ZJ. et al., 2021; Du et al., 2021). Anticancer activities are increased by iron treatment, including ferrous iron or transferrin (Jin et al., 2023). Compared with normal cells, cancer cells possess high intracellular iron levels, which support their growth (HaSsannia et al., 2019). Osteoclasts share iron metabolism characteristics with cancer cells, making them more susceptible to iron-induced ferroptosis. Our findings revealed that Ssa increased lipid peroxidation and induced ferroptotic cell mitochondrial features. The induction of ferroptosis might be associated with the iron-mediated activation of Ssa. Our data suggest that iron enhances osteoclast lipid peroxidation, leading to ferroptosis, thus inhibiting the differentiation of osteoclasts.

Reducing oxidative stress has been shown to be beneficial in many pathological lytic bone disorders (Zhang et al., 2023). However, Jin et al. (2023) revealed that Artesunate inhibits osteoclast differentiation by inducing ferroptosis and prevents iron overload-induced bone loss. In addition, the observations of Yang et al. (Yang et al., 2022) indicated that targeting ferroptosis could suppress osteocyte glucolipotoxicity and alleviate diabetic osteoporosis. Our study confirmed that Ssa could generate excessive oxidative stress, thereby attenuating osteoclastogenesis by increasing ferroptosis.

In recent studies, ferroptosis was suggested to be a type of necrotic death in which damage-associated molecular patterns are released, leading to increased infiltration of immune cells and an inflammatory response (Li et al., 2019; Sun Y. et al., 2020). However, other research has proposed that ferroptosis is a physiologically therapeutic process involving anti neoplastic and anti-inflammatory effects (Ebel et al., 2014; Arbiser et al., 2018). In this study, Ssa attenuated *in vitro* osteoclastogenesis and decreased the bone loss induced by LPS *in vivo* by promoting osteoclast ferroptosis, suggesting that the inflammatory response of bone loss induced by LPS involves ferroptosis. However, further research is needed to pinpoint the molecular pathways that link periodontal inflammation with ferroptosis. The apparently contradictory results mentioned above reinforce the need to further investigate the correlation between inflammation and ferroptosis in lytic bone disorders.

Although Ssa inhibited *in vitro* osteoclastogenesis and decreased *in vivo* LPS induced bone loss by promoting osteoclasts ferroptosis, the active ingredients in Ssa extracts are unknown. In a future study, we will determine Ssa’s active ingredients, which will allow investigation their direct mechanisms. Moreover, *in vitro*, Ssa caused BMM toxicity at concentrations greater12.5 μM. Therefore, Ssa cytotoxicity should be taken into account for future clinical application.

## 5 Conclusion

Ssa could inhibit osteoclastogenesis *in vitro*, and attenuated LPS-mediated bone loss *in vivo* by promoting osteoclast ferroptosis. This promotion of ferroptosis occurred, at least in part, by attenuating Nrf2/SLC7A11/GPX4 signaling. Thus, Ssa represents a potential therapeutic approach for a series of pathological lytic bone disorders.

## Data Availability

The original contributions presented in the study are included in the article/Supplementary Material, further inquiries can be directed to the corresponding author.

## References

[B1] Alborzinia H. Flórez A. F. Kreth S. Brückner L. M. Yildiz U. Gartlgruber M. (2022). MYCN mediates cysteine addiction and sensitizes neuroblastoma to ferroptosis. Nat. Cancer 3 (4), 471–485. 10.1038/s43018-022-00355-4 35484422 PMC9050595

[B2] Arbiser J. L. Bonner M. Y. Ward N. Elsey J. Rao S. (2018). Selenium unmasks protective iron armor: a possible defense against cutaneous inflammation and cancer. Biochim. Biophys. Acta Gen. Subj. 1862 (11), 2518–2527. 10.1016/j.bbagen.2018.05.018 29852199 PMC6934939

[B3] Ashour M. L. Wink M. (2011). Genus Bupleurum: a review of its phytochemistry, pharmacology and modes of action. J. Pharm. Pharmacol. 63 (3), 305–321. 10.1111/j.2042-7158.2010.01170.x 21749378 PMC7197585

[B4] Bertuglia A. Lacourt M. Girard C. Beauchamp G. Richard H. Laverty S. (2016). Osteoclasts are recruited to the subchondral bone in naturally occurring post-traumatic equine carpal osteoarthritis and may contribute to cartilage degradation. Osteoarthr. Cartil. 24 (3), 555–566. 10.1016/j.joca.2015.10.008 26505663

[B5] Boyle W. J. Simonet W. S. Lacey D. L. (2003). Osteoclast differentiation and activation. Nature 423 (6937), 337–342. 10.1038/nature01658 12748652

[B6] Chang S. Tang M. Zhang B. Xiang D. Li F. (2022). Ferroptosis in inflammatory arthritis: a promising future. Front. Immunol. 13, 955069. 10.3389/fimmu.2022.955069 35958605 PMC9361863

[B7] Chen G. Q. Benthani F. A. Wu J. Liang D. Bian Z. X. Jiang X. (2020). Artemisinin compounds sensitize cancer cells to ferroptosis by regulating iron homeostasis. Cell Death Differ. 27 (1), 242–254. 10.1038/s41418-019-0352-3 31114026 PMC7205875

[B8] Chen X. Yu C. Kang R. Kroemer G. Tang D. (2021). Cellular degradation systems in ferroptosis. Cell Death Differ. 28 (4), 1135–1148. 10.1038/s41418-020-00728-1 33462411 PMC8027807

[B9] Compston J. E. McClung M. R. Leslie W. D. (2019). Osteoporosis. Lancet. 393 (10169), 364–376. 10.1016/S0140-6736(18)32112-3 30696576

[B10] Dixon S. J. Lemberg K. M. Lamprecht M. R. Skouta R. Zaitsev E. M. Gleason C. E. (2012). Ferroptosis: an iron-dependent form of nonapoptotic cell death. Cell 149 (5), 1060–1072. 10.1016/j.cell.2012.03.042 22632970 PMC3367386

[B11] Du J. Wang X. Li Y. Ren X. Zhou Y. Hu W. (2021). DHA exhibits synergistic therapeutic efficacy with cisplatin to induce ferroptosis in pancreatic ductal adenocarcinoma via modulation of iron metabolism. Cell Death Dis. 12 (7), 705. 10.1038/s41419-021-03996-y 34262021 PMC8280115

[B12] Du Z.-A. Sun M.-N. Hu Z.-S. (2018). Saikosaponin a ameliorates LPS-induced acute lung injury in mice. Inflammation 41 (1), 193–198. 10.1007/s10753-017-0677-3 28986747

[B13] Ebel P. Imgrund S. Vom Dorp K. Hofmann K. Maier H. Drake H. (2014). Ceramide synthase 4 deficiency in mice causes lipid alterations in sebum and results in alopecia. Biochem. J. 461 (1), 147–158. 10.1042/BJ20131242 24738593

[B14] Fu Y. Hu X. Cao Y. Zhang Z. Zhang N. (2015). Saikosaponin a inhibits lipopolysaccharide-oxidative stress and inflammation in Human umbilical vein endothelial cells via preventing TLR4 translocation into lipid rafts. Free Radic. Biol. Med. 89, 777–785. 10.1016/j.freeradbiomed.2015.10.407 26475038

[B15] Gao H. Song Y. Li D. Feng W. Liu J. (2017). Saikosaponin A inhibits IL-1β-induced inflammatory mediators in human osteoarthritis chondrocytes by activating LXRα. Oncotarget 8 (51), 88941–88950. 10.18632/oncotarget.21495 29179489 PMC5687659

[B16] Gao M. Fan K. Chen Y. Zhang G. Chen J. Zhang Y. (2022). Understanding the mechanistic regulation of ferroptosis in cancer: the gene matters. J. Genet. Genomics 49 (10), 913–926. 10.1016/j.jgg.2022.06.002 35697272

[B17] Hadji P. Papaioannou N. Gielen E. Feudjo Tepie M. Zhang E. Frieling I. (2015). Persistence, adherence, and medication-taking behavior in women with postmenopausal osteoporosis receiving denosumab in routine practice in Germany, Austria, Greece, and Belgium: 12-month results from a European non-interventional study. Osteoporos. Int. 26 (10), 2479–2489. 10.1007/s00198-015-3164-4 26018090 PMC4575374

[B18] HaSsannia B. Vandenabeele P. Vanden Berghe T. (2019). Targeting ferroptosis to iron out cancer. Cancer Cell 35 (6), 830–849. 10.1016/j.ccell.2019.04.002 31105042

[B19] Hirschhorn T. Stockwell B. R. (2019). The development of the concept of ferroptosis. Free Radic. Biol. Med. 133, 130–143. 10.1016/j.freeradbiomed.2018.09.043 30268886 PMC6368883

[B20] Hough F. S. Brown S. L. Cassim B. Davey M. R. de Lange W. de Villiers T. J. (2014). The safety of osteoporosis medication. S Afr. Med. J. 104 (4), 279–282. 10.7196/samj.7505 25118550

[B21] Jiang X. Stockwell B. R. Conrad M. (2021). Ferroptosis: mechanisms, biology and role in disease. Nat. Rev. Mol. Cell Biol. 22 (4), 266–282. 10.1038/s41580-020-00324-8 33495651 PMC8142022

[B22] Jin Y. Wu S. Zhang L. Yao G. Zhao H. Qiao P. (2023). Artesunate inhibits osteoclast differentiation by inducing ferroptosis and prevents iron overload-induced bone loss. Basic Clin. Pharmacol. Toxicol. 132 (2), 144–153. 10.1111/bcpt.13817 36433916

[B23] Kapasa E. R. Giannoudis P. V. Jia X. Hatton P. V. Yang X. B. (2017). The effect of RANKL/OPG balance on reducing implant complications. J. Funct. Biomater. 8 (4), 42. 10.3390/jfb8040042 28937598 PMC5748549

[B24] Kim B. M. (2018). The role of saikosaponins in therapeutic strategies for age-related diseases. Oxid. Med. Cell Longev. 2018, 8275256. 10.1155/2018/8275256 29849917 PMC5924972

[B25] Koehn F. E. Carter G. T. (2005). The evolving role of natural products in drug discovery. Nat. Rev. Drug Discov. 4 (3), 206–220. 10.1038/nrd1657 15729362

[B26] Li N. Cornelissen D. Silverman S. Pinto D. Si L. Kremer I. (2021b). An updated systematic review of cost-effectiveness analyses of drugs for osteoporosis. Pharmacoeconomics 39 (2), 181–209. 10.1007/s40273-020-00965-9 33026634 PMC7867562

[B27] Li N. Jiang W. Wang W. Xiong R. Wu X. Geng Q. (2021a). Ferroptosis and its emerging roles in cardiovascular diseases. Pharmacol. Res. 166, 105466. 10.1016/j.phrs.2021.105466 33548489

[B28] Li W. Feng G. Gauthier J. M. Lokshina I. Higashikubo R. Evans S. (2019). Ferroptotic cell death and TLR4/Trif signaling initiate neutrophil recruitment after heart transplantation. J. Clin. Invest. 129 (6), 2293–2304. 10.1172/JCI126428 30830879 PMC6546457

[B29] Li Z. J. Dai H. Q. Huang X. W. Feng J. Deng J. H. Wang Z. X. (2021c). Artesunate synergizes with sorafenib to induce ferroptosis in hepatocellular carcinoma. Acta Pharmacol. Sin. 42 (2), 301–310. 10.1038/s41401-020-0478-3 32699265 PMC8026986

[B30] Lin M. T. Beal M. F. (2006). Mitochondrial dysfunction and oxidative stress in neurodegenerative diseases. Nature 443 (7113), 787–795. 10.1038/nature05292 17051205

[B31] Mukherjee K. Chattopadhyay N. (2016). Pharmacological inhibition of cathepsin K: a promising novel approach for postmenopausal osteoporosis therapy. Biochem. Pharmacol. 117, 10–19. 10.1016/j.bcp.2016.04.010 27106079

[B32] Pihlstrom B. L. Michalowicz B. S. Johnson N. W. (2005). Periodontal diseases. Lancet. 366 (9499), 1809–1820. 10.1016/S0140-6736(05)67728-8 16298220

[B33] Qiao S. Li B. Cai Q. Li Z. Yin Z. He J. (2022). Involvement of ferroptosis in Porphyromonas gingivalis lipopolysaccharide-stimulated periodontitis *in vitro* and *in vivo* . Oral Dis. 29, 3571–3582. 10.1111/odi.14292 35765229

[B34] Rachner T. D. Khosla S. Hofbauer L. C. (2011). Osteoporosis: now and the future. Lancet 377 (9773), 1276–1287. 10.1016/S0140-6736(10)62349-5 21450337 PMC3555696

[B35] Stockwell B. R. (2022). Ferroptosis turns 10: emerging mechanisms, physiological functions, and therapeutic applications. Cell. 185 (14), 2401–2421. 10.1016/j.cell.2022.06.003 35803244 PMC9273022

[B36] Sun X. Xie Z. Hu B. Zhang B. Ma Y. Pan X. (2020a). The Nrf2 activator RTA-408 attenuates osteoclastogenesis by inhibiting STING dependent NF-κb signaling. Redox Biol. 28, 101309. 10.1016/j.redox.2019.101309 31487581 PMC6728880

[B37] Sun Y. Cai T. T. Zhou X. B. Xu Q. (2009). Saikosaponin a inhibits the proliferation and activation of T cells through cell cycle arrest and induction of apoptosis. Int. Immunopharmacol. 9 (7-8), 978–983. 10.1016/j.intimp.2009.04.006 19375524

[B38] Sun Y. Chen P. Zhai B. Zhang M. Xiang Y. Fang J. (2020b). The emerging role of ferroptosis in inflammation. Biomed. Pharmacother. 127, 110108. 10.1016/j.biopha.2020.110108 32234642

[B39] Takayanagi H. (2007). Osteoimmunology: shared mechanisms and crosstalk between the immune and bone systems. Nat. Rev. Immunol. 7 (4), 292–304. 10.1038/nri2062 17380158

[B40] Tang D. Chen X. Kang R. Kroemer G. (2021). Ferroptosis: molecular mechanisms and health implications. Cell Res. 31 (2), 107–125. 10.1038/s41422-020-00441-1 33268902 PMC8026611

[B41] Xie L. W. Cai S. Zhao T. S. Li M. Tian Y. (2020). Green tea derivative (-)-epigallocatechin-3-gallate (EGCG) confers protection against ionizing radiation-induced intestinal epithelial cell death both *in vitro* and *in vivo* . Free Radic. Biol. Med. 161, 175–186. 10.1016/j.freeradbiomed.2020.10.012 33069855

[B42] Yang Y. Lin Y. Wang M. Yuan K. Wang Q. Mu P. (2022). Targeting ferroptosis suppresses osteocyte glucolipotoxicity and alleviates diabetic osteoporosis. Bone Res. 10 (1), 26. 10.1038/s41413-022-00198-w 35260560 PMC8904790

[B43] Yuan Y. Zhai Y. Chen J. Xu X. Wang H. (2021). Kaempferol ameliorates oxygen-glucose deprivation/reoxygenation-induced neuronal ferroptosis by activating nrf2/slc7a11/GPX4 Axis. Biomolecules 11 (7), 923. 10.3390/biom11070923 34206421 PMC8301948

[B44] Zaidi M. (2007). Skeletal remodeling in health and disease. Nat. Med. 13 (7), 791–801. 10.1038/nm1593 17618270

[B45] Zhang C. Li H. Li J. Hu J. Yang K. Tao L. (2023). Oxidative stress: a common pathological state in a high-risk population for osteoporosis. Biomed. Pharmacother. 163, 114834. 10.1016/j.biopha.2023.114834 37163779

[B46] Zhang Y. Swanda R. V. Nie L. Liu X. Wang C. Lee H. (2021). mTORC1 couples cyst(e)ine availability with GPX4 protein synthesis and ferroptosis regulation. Nat. Commun. 12 (1), 1589. 10.1038/s41467-021-21841-w 33707434 PMC7952727

[B47] Zhao T. Yang Q. Xi Y. Xie Z. Shen J. Li Z. (2022). Ferroptosis in rheumatoid arthritis: a potential therapeutic strategy. Front. Immunol. 13, 779585. 10.3389/fimmu.2022.779585 35185879 PMC8847160

[B48] Zhou C. Liu W. He W. Wang H. Chen Q. Song H. (2015). Saikosaponin a inhibits RANKL-induced osteoclastogenesis by suppressing NF-κB and MAPK pathways. Int. Immunopharmacol. 25 (1), 49–54. 10.1016/j.intimp.2015.01.010 25617149

