# Supplementary figures and images for: Saikosaponin A attenuates osteoclastogenesis and bone loss by inducing ferroptosis
Source: Front Mol Biosci. 2024 Jul 24;11:1390257. doi: 10.3389/fmolb.2024.1390257 (PMC11303733; doi:10.3389/fmolb.2024.1390257)

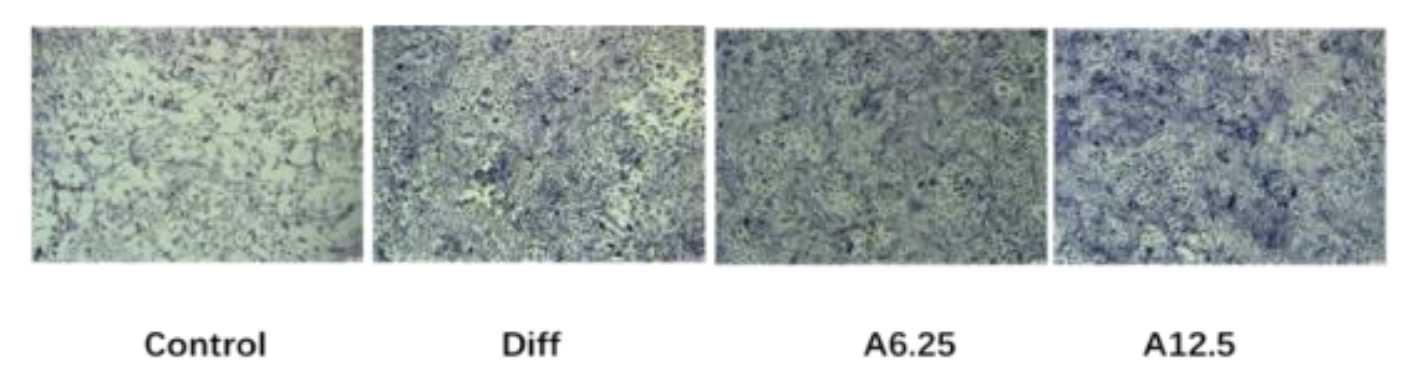

Supplement: Supplementary file 1 [file Image1.png]
